# Optimization of Selected Minerals and a Cytokinin for *In Vitro* Propagation of Little-Leaf Mockorange (*Philadelphus microphyllus* A. Gray) Using Response Surface Methodology (RSM)

**DOI:** 10.3390/plants13233446

**Published:** 2024-12-09

**Authors:** Razieh Khajehyar, Robert Tripepi, William J. Price, Stephen Love

**Affiliations:** 1 Department of Plant Sciences, University of Idaho, Moscow, ID 83843, USA; 2 Department of Mathematics and Statistical Sciences, University of Idaho, Moscow, ID 83843, USA; 3 Department of Plant Sciences, Aberdeen Research and Extension Center, University of Idaho, Aberdeen, ID 83210, USA

**Keywords:** tissue culture, statistical modeling, axillary shoot culture, culture medium optimization, native landscape plants, sustainable horticulture

## Abstract

Optimizing concentrations of minerals and phytohormones is essential when culturing a new plant species. The objective of this study was to use Response Surface Methodology (RSM) to evaluate combinations of selected minerals (N, Ca, and P) along with zeatin (Zea) to obtain optimum shoot growth of little-leaf mockorange. Forty-six treatment combinations were assigned using Proc Optex in SAS software version 9.4. The concentrations of Zea tested were 0.82, 1.095, or 1.37 µM, and the minerals were 22.5, 30, or 37.5 mM N, 1.13, 1.5, or 1.875 mM Ca, and 0.31, 0.625, or 0.937 mM P. Treatment concentrations were tested for their effects on the number of axillary shoots formed, shoot length, and dry weight. The response surface analyses showed that the optimum concentrations of N, Ca, and P were 34 to 39 mM, 1.5 mM, and 0.625 mM, respectively. Medium supplemented with 1.1 µM Zea affected shoot growth positively. Comparison of mineral concentrations in medium with concentrations in full-strength Murashige and Skoog (MS) medium, suggests ½ MS medium should be appropriate to efficiently multiply little-leaf mockorange shoots efficiently, thus saving the time and money involved in creating a custom medium formulation.

## 1. Key Message

Optimal concentrations of mineral ingredients and Zea in tissue culture medium used for proliferation of *Philadelphus microphyllus* (little-leaf mockorange) were determined using Response Surface Methodology (RSM). Results demonstrated that concentrations of evaluated minerals equivalent to ½ strength MS medium were ideal for improving the shoot growth of micropropagated little-leaf mockorange.

## 2. Introduction

Plant response to tissue culture propagation is highly dependent on the presence and amount of minerals and nutrients supplied in culture media, including nitrogen (N) [1], potassium (K), calcium (Ca), phosphorous (P), magnesium (Mg), and iron (Fe). Such nutrients play key roles in plant growth and development due to involvement in structural or metabolic processes. Hence, the mineral concentration and ratios of various minerals within a culture medium impact aspects of plant morphogenesis, such as shoot multiplication and elongation, and conversely may contribute to abnormal growth or physiological disorders [2,3]. Similarly, *in vitro* plant growth regulators (PGRs) may either enhance growth or may also produce physiological disorders, depending on type and concentration [2]. Shoot explants of raspberries grown on culture media without or containing low concentrations of auxins or cytokinins grew poorly on culture media and began to senesce. Changes in mineral concentrations in culture media are interactive and may affect the amount of uptake of other minerals as well, thus, affecting plant growth and development [4].

Murashige and Skoog (MS) [5] salt formulation is a common, commercially available source of minerals employed to grow many plant species *in vitro*. This salt formulation may be suboptimal for some species, causing them to grow slowly [6]. Red raspberry shoots grew poorly *in vitro* on MS medium [4]. Small adjustments in commercially available medium mineral and phytohormone content may enhance plant growth [6]. Even individual cultivars within a species may differ in their response to different mineral combinations, meaning genotype-specific formulations of a culture medium can be highly useful for developing optimized *in vitro* propagation procedures for some plant species [7].

In plant tissue culture, the growth response of explants can vary significantly depending on the plant species and specific medium composition used. While Murashige and Skoog (MS) medium is widely utilized for its balanced nutrient profile, several studies have reported suboptimal growth of certain plant species on standard MS medium. For example, *Eucalyptus camaldulensis* explants showed reduced growth on full-strength MS medium; diluting the medium to half-strength improved their development, suggesting that high salt concentrations in full-strength MS medium can inhibit growth in some species [8]. Similarly, in a study on Eucalyptus hybrid clones (*Eucalyptus grandis* × *E. urophylla*), researchers found that the Woody Plant Medium (WPM) supported better growth compared to MS medium due to its lower salt concentration, which proved more suitable for these clones [9]. General reviews and studies, such as those by Phillips and Garda [10], have emphasized that MS medium may require modification for optimal results with certain species, as nutritional needs differ widely across plant types. Additionally, Mariusz Gaj [11] has highlighted that adjustments in hormone concentrations and nutrient levels are often necessary, as standard MS medium may not fully support optimal growth for all species. These findings underline the importance of customizing tissue culture media based on the physiological needs of the species in question to achieve enhanced growth outcomes.

Developing optimized medium formulations compatible for a specific cultivar is a complicated and time-consuming process [7]. Necessity for changing the concentration and combination of different organic compounds or minerals, as well as different PGRs needed by explants during each of the progressive phases of tissue culture, complicates the process further. Completing experiments to find the best concentration of each individual mineral can take months, but combining all individual experiments into one by applying factorial combinations of nutrients and PGRs can reduce the time requirement. The traditional method of optimizing the tissue culture medium was to conduct a series of trials wherein individual ingredients were tested at a range of concentrations [4]. Once that ingredient concentration was optimized, trials would begin for subsequent medium individual components. MS medium was developed in this way specifically for use in producing tobacco callus cells and, subsequently, has been used for culturing many plant species. Not only is this systematic medium development process inefficient, it fails to account for concentration-dependent interactions among ingredients.

Although evaluation of all possible combinations of a large number of components can be logistically challenging or impossible, statistical tools are available to generate experimental designs capable of estimating optimal component concentrations without the need for serially arranged experiments [6,12]. These experimental designs provide more flexibility and efficiency to improve tissue culture media for specific plant species [7,12]. Using RSM, linear and quadratic surface models were generated using data collected within the appropriate design. Given adequate model fit, optimal mineral and PGR values can then be algebraically derived from the estimated model. Wada et al. [1,13] conducted a series of studies using different mineral compounds in pear tissue culture medium across five different genotypes. Niedz and Evens [12] used RSM to develop tissue culture medium for pear shoots and found that the mesos compounds (CaCl_2_, MgSO_4_, and KH_2_PO_4_) as supplements in MS medium were important growth factors for pear shoot cultures. Among other things, these researchers demonstrated that varying minerals in the culture medium simultaneously is more efficient than examining only one mineral at a time [1].

Response Surface Methodology has also recently been used to model and optimize *in vitro* propagation medium by interpreting each medium component or important minerals or PGRs as a geometric dimension, which results in a geometric volume having *n* dimensions [4,6,7,12].

Assorted researchers have used RSM to develop and optimize the components of tissue culture media and enhance the proliferation and shoot regeneration of plant species. Documented examples include: improving somatic embryogenesis and regeneration of henbane (*Hyoscyamus niger*) by optimizing the concentrations of PGRs, such as auxins and cytokinins using RSM [14]; optimizing the PGRs and silver nitrate for micropropagation of carnation (*Dianthus caryophyllus* L.) using RSM [15]; optimizing media for establishment and proliferation culture phases for Iranian seedless barberry (*Berberis vulgaris* L. var. *asperma*) [16]; optimization of a shoot regeneration protocol in basilicum (*Basilicum polystachyon*) [17]; and optimizing the culture medium to optimize the embryogenic callus induction of sugarcane (*Saccharum* sp.) [18]. Researchers have also applied RSM to evaluate types of culture induction tissue in soybean (*Glycine max* L. Merr) callus productions and tissue regeneration [19].

In RSM, geometric dimensions refer to the shape, size, or arrangement of the experimental factors or variables that are being studied. Geometric dimensions in RSM involve adjusting the levels or settings of the independent variables (factors) in a way that allows for the exploration of the relationship between these factors and the response. The geometric volume provided by the statistical analysis software is considered as the sample space for the experimental design that contains the samples (treatment combinations) based on the objectives of the experiment. Results obtained from data collection of the dependent variables, such as shoot growth characteristics, could help determine the optimum concentrations of each component in an optimized medium [7]. With the help of computer-aided, statistical design software, different formulations of culture media are assigned. These points (treatments) are then applied and evaluated based on the plant responses of interest (dependent variables) to the treatments to create a multi-dimensional response surface. This information is applied to a response surface model that is used to determine the optimal plant response and corresponding treatments [7].

Little-leaf mockorange (*Philadelphus microphyllus* A. Gray) is native to the western United States and has potential for use in managed urban landscapes. This species is very tolerant to drought, easy to grow, and has minimal maintenance requirements. It can also grow in a wide range of light conditions from light shade to full sun [20,21,22]. Altogether, the characteristics of this plant species make it ideal for use in western landscapes. Many of the mockorange species can be propagated by seeds, summer soft-wood cuttings, hardwood cuttings and layering, and little-leaf mockorange produces highly variable progeny from seed and is difficult to propagate using cuttings [23]. However, information about propagating this specific native species is lacking in the literature. A viable *in vitro* propagation system would enhance the value of the attractive, landscape-worthy species.

The objectives of this study were to optimize a tissue culture media for little-leaf mockorange shoots by using RSM with selected medium components. We proposed to evaluate important minerals, such as nitrogen (N), phosphorus (P), calcium (Ca), and potassium (K), as well as zeatin (Zea), across a range of concentrations. The responses obtained from the experiment will determine the best combination of the selected components for producing optimum growth of *in vitro* shoots of little-leaf mockorange as defined by maximized shoot number, length, and shoot biomass.

## 3. Results

Results of RSM modeling are presented as predicted multiple design treatment points, hence, interpretation was often completed through graphical representations presented as the estimated responses over the continuum of component concentrations [7]. Considering components two at a time, results are displayed two-dimensionally as a series of contour plots for each response variable. If the design points encompass an optima, the optimum value of each factor was determined to be somewhere in the center of the contour surface, represented by a circular or elliptical zone. Any value outside this zone indicates the dependent variable, e.g., shoot growth, failed to respond optimally (grow well) to the imposed treatments.

Although K was evaluated in this study, this element in the regression models resulted in nonsignificant changes in responses. Hence, K concentrations were limited to linear trends in the analyses, and this mineral was eliminated from further RSM analysis.

### 3.1. Number of Axillary Shoots

**Figure 1:**
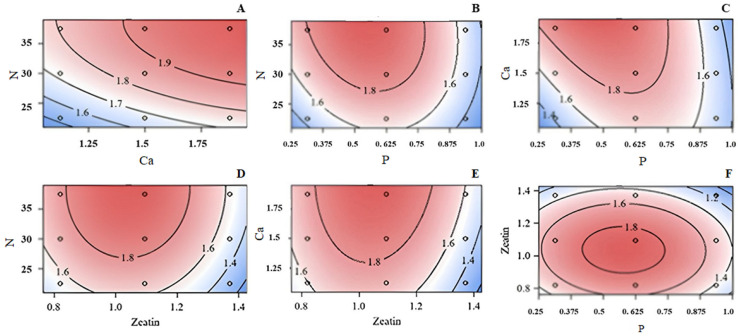
Response surface of concentrations of N (mM) versus Ca (mM) (**A**), N (mM) versus P (mM) (**B**), Ca versus P (**C**), N (mM) versus Zea (μM) (**D**), Ca (mM) versus N (mM) (**E**), or Zea (μM) versus P (mM) (**F**) on axillary shoot number for little-leaf mockorange shoots produced *in vitro*. Blue color indicated poor growth and red demonstrated the optimum growth. Values on contour lines indicate the level of response (mean axillary shoot number).

Regression analysis of the response surface for the number of explant axillary shoots, showed that both linear and quadratic regressions were highly significant (*p* = 0.0081 and 0.0005, respectively), whereas the crossproduct terms were nonsignificant. Response surfaces were created for pairs of mineral and PGR concentrations, such as Ca vs. N (Figure 1A), P vs. N (Figure 1B), P vs. Ca (Figure 1C), Zea vs. N (Figure 1D), Zea vs. Ca (Figure 1E), and P vs. Zea (Figure 1F). Both Ca and N revealed open contour lines for shoot numbers, with maximum responses at the upper limits of the concentrations evaluated. The open contours indicated the optimal shoot growth for these minerals may occur at or higher than the highest tested levels or higher. The relationship between Zea and P concentrations, however, was stronger and more definitive (Figure 1F). The complete circle surface revealed that shoot growth was optimized toward the center of the enclosed zone. Overall, the optimum number of axillary shoots resulted from a combination of 0.6 mM P and 1.1 µM Zea in the culture medium.

The optimum concentrations for each medium component N, Ca, P, and Zea were 39.28 mM, 2.95 mM, 0.25 mM, and 1.09 µM, respectively. Potassium was assumed to be constant at 10 mM. The predicted response value at this point (optimum point) was estimated as 2.2 axillary shoots per explant (Figure 1).

### 3.2. Shoot Length

**Figure 2:**
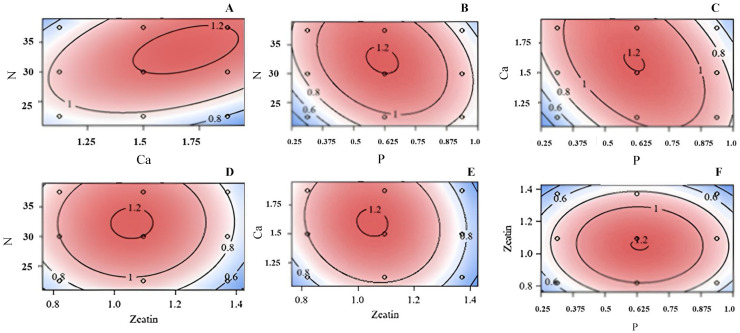
Response surface of concentrations of N (mM) versus Ca (mM) (**A**), N (mM) versus P (mM) (**B**), Ca versus P (**C**), N (mM) versus Zea (μM) (**D**), Ca (mM) versus N (mM) (**E**), or Zea (μM) versus P (mM) (**F**) on shoot length for little-leaf mockorange shoots produced *in vitro*. Blue color indicated poor growth and red demonstrated the optimum growth. Values on contour lines indicate the level of response (mean shoot length (cm)).

Response Surfaces were generated for shoot length across concentrations of Ca vs. N (Figure 2A), P vs. N (Figure 2B), P vs. Ca (Figure 2C), Zea vs. N (Figure 2D), Zea vs. Ca (Figure 2E), and P vs. Zea (Figure 2F). Most paired factors exhibited a strong relationship as indicated by the enclosed contours of these figures. Enclosed contours represent areas where specific combinations of factors, such as concentrations of Ca, N, P, and Zea, resulted in shoot lengths that were close or identical, meaning that within these regions of the graphs, different combinations of these factors produced similar effects on shoot length, indicating a consistent relationship between the variables.

Response Surface analysis showed that among the regression models only a quadratic regression was significant for the shoot length of *in vitro* little-leaf mockorange, (*p* < 0.0001). The optimum concentrations for N, Ca, P, and Zea were estimated as 36.66 mM, 1.89 mM, 0.5 mM, and 1.04 µM, respectively, and the predicted shoot length at the stationary point (optimum) of shoot length was 1.3 cm.

### 3.3. Dry Weight

**Figure 3:**
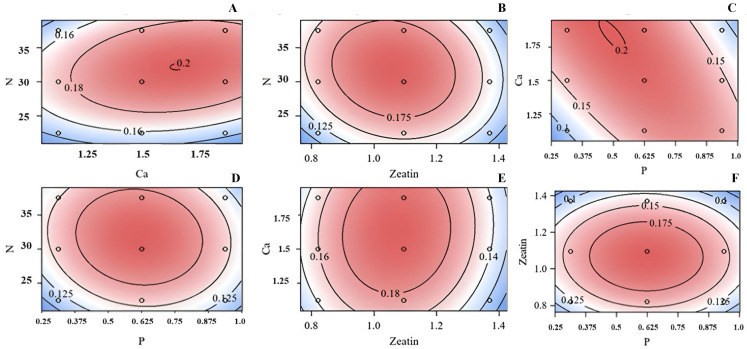
Response surface of concentrations of N (mM) versus Ca (mM) (**A**), N (mM) versus P (mM) (**B**), Ca versus P (**C**), N (mM) versus Zea (μM) (**D**), Ca (mM) versus N (mM) (**E**), or Zea (μM) versus P (mM) (**F**) on shoot dry weight (g) for little-leaf mockorange shoots produced *in vitro*. Blue color indicated poor growth and red demonstrated the optimum growth. Values on contour lines indicate the level of response (mean shoot dry weight (g)).

Response surfaces for shoot dry weight were determined for concentrations of Ca vs. N (Figure 3A), Zea vs. N (Figure 3B), P vs. Ca (Figure 3C), P vs. N (Figure 3D), Zea vs. Ca (Figure 3E), and P vs. Zea (Figure 3F). A strong relationship was demonstrated between most mineral components and Zea as indicated by the complete contours in the graphical representations. The center of each zone represented the optimum concentration for each selected variable (minerals or Zea).

For explant dry weight, only the quadratic regression was significant (*p* = 0.0001). The optimum concentrations for N, Ca, P, and Zea were calculated as 34.08 mM, 2.0 mM, 0.41 mM, and 1.1 µM, respectively, whereas the predicted dry weight at the stationary point (optimum) was 0.21 mg. Little-leaf mockorange shoots grew well and produced the most dry weight within the range of minerals and Zea included within the design of this experiment.

## 4. Discussion

Optimal concentrations of minerals and plant growth regulators in the tissue culture media are critical for efficacious shoot multiplication. Murashige and Skoog (MS) medium contains all minerals necessary for many plant species. However, many species require modifications to MS basal salts for ideal growth. Finding the optimum amount of each mineral or PGR to include in tissue culture media can be time-consuming and expensive, even without considering failed experiments. Response Surface Methodology is one of the fastest methods of coincidentally testing independent variables in biological experiments [24]. A number of studies have been completed using RSM to modify culture media formulations for different plant species, such as determining mineral concentrations for efficient propagation of red raspberry [4,6], modifying the mesos components concentrations in culture medium for pear [13], improving the culture medium for micropropagation of pear germplasm [7], and optimizing nitrate and ammonium requirements as well as nitrogen concentrations needed for pear shoot cultures [1,3].

Researchers verify that RSM can be applied with high precision in biological study models, using minimal resources while maintaining excellent efficiency [19]. This method offers two key advantages over traditional factorial designs: (1) RSM allows researchers to study the effects of independent variables between actual experimental data points, and (2) RSM enables the inclusion of more than five experimental variables, which is typically impractical in standard factorial designs [25].

Premkumar et al. [26] used RSM to optimize concentrations of kinetin, indoleacetic acid (IAA), 6-benzylaminopurine (BAP), or sucrose for liquid culture shoot regeneration and enriched levels of scopadulcic acid B in leaf cultures of *Scoparia dulcis* L. Another successful study employing RSM to predict and optimize the cell suspension culture was reported by Farjaminezhad and Garoosi [27] applying the RSM to optimize the suspension culture of *Azadirachta indica*. Bagherieh-Najjar and Nezamdoost [25] employed RSM to optimize values of BAP and IAA for callus production and shikonin production by *O. dichroantha* on White medium. However, they failed to provide enclosed desirability contour plots for callus production of the same species on B5 and M9 medium. Although Bansal et al. [28] increased cell growth and bacoside-A production in suspension cultures of *Bacopa monnieri* L Wettst, by applying RSM, their study failed to determine the exact and optimum value of the glucose or KNO_3_ concentrations in the culture medium.

Unlike other tissue culture studies, most RSM response surfaces created in this study resulted in closed contours with a clear optimum point. Closed contours in the response surfaces allowed clear identification of the optimum concentration for each component applied in the experiment. A complete circle or ellipse on the surface response confirmed that the ranges of the applied components in the experiment were selected appropriately with optimums falling within the experimental parameters.

Notably, the enclosed contours within the figures presented in this study served as visual indicators of the robust relationships between the fixed variables and the response variables, such as shoot length. Enclosed contours signify regions where specific combinations of factors result in similar outcomes for shoot length. The prominence of enclosed contours across the figures indicated a strong correlation between the fixed variables and the response variable, underscoring the significance of these factors in influencing shoot length. This observation enhanced our understanding of how changes in concentrations of Ca, N, P, and Zea impact shoot length, thereby providing valuable insights for optimizing growth conditions and enhancing shoot growth.

Based on these results, optimum concentrations of Zea, N, Ca, or P for maximizing shoot proliferation of little-leaf mockorange were identified (Table 1). Results of the axillary shoot numbers formed and showed that to obtain more axillary shoots and obtain a complete circle on the response surface, the concentrations of N and Ca probably needed to be increased.

## 5. Materials and Methods

### 5.1. Plant Materials

In 2019, stems from little-leaf mockorange (*Philadelphus microphyllus*) were collected from plots located at the University of Idaho Aberdeen Research and Extension Center, Aberdeen, Idaho, USA. Samples were shipped to the Plant Tissue Culture Lab in the Plant Sciences Department at University of Idaho, Moscow, Idaho, USA. Provenance of the source plant was collected from the Goshutes Mountains, south of Wendover in Elko County, Nevada (collected in 2012 by Dr. Stephen Love).

### 5.2. Micropropagation and Maintenance of the Shoot Cultures

Shoot cultures were established and then maintained and stabilized on culture medium for at least 6 months and were subcultured monthly. Each subculture cycle was completed by cutting the stems into 1.5 cm pieces with each explant consisting of two to three nodes. Six stem explants were placed into each baby food jar (195 mL). Explants were incubated in an SG-30S germinator (Hoffman Manufacturing Inc., Albany, OR, USA) at 25 ± 1 °C under a 16 h photoperiod (cool-white fluorescent lamps), with 38 μmol·m^−2^·s^−1^ photosynthetic photon flux (PPF). Stable shoot cultures were used in all the following experiments.

### 5.3. Response Surface Methodology Experiment

#### Experimental Design

The RSM experimental design was created using Proc Optex procedure [29]. This method created an orthogonal balanced incomplete block design (Table 2). A total of 46 treatments were included in the design. The algorithm used predetermined high and low values for each nutrient and then set the middle level as an average based on these values (Table 3). High and low values for each mineral were determined from preliminary observation and experimentation [23]. The overall experimental design was a five-factor Response Surface design with the design points (combinations of all five factors) selected using modified D-optimal criteria suitable for fitting a quadratic polynomial equation. This experiment was completed for stage II of axillary shoot micropropagation.

### 5.4. Media Preparation and Micropropagation

All media contained the standard amounts of ½ MS medium including 9 mg MgSO_4_, 1.4 mg FeSO_4_.7H_2_O, 1.86 mg Na_2_EDTA.2H_2_O, 0.85 mg MnSO_4_.H_2_O, 0.43 mg ZnSO_4_.7H_2_O, 0.31 mg H_3_BO_3_, 0.012 mg Na_2_MoO_4_.2H_2_O, 0.001 mg CuSO_4_.5H_2_O, 0.001 mg CoCl.6H_2_O, 3 g sucrose, 0.05 mg thiamine-HCl, 0.025 mg nicotinic acid, 0.025 mg pyridoxine-HCl, 0.1 mg glycine, and 0.005 g myo-inositol, as well as 0.35 g agar in 100 mL. To compensate for the extra nitrogen, potassium, sulphate, phosphate, or chloride contents required in the original ½ MS medium, NaNO_3_, K_2_SO_4_, NaCl, or NaH_2_PO_4_ were added to the specific treatment medium based on the concentrations of the needed components. Adding these minerals helped to offset the lost necessary components in the media (Supplementary Materials).

Medium pH was adjusted to 5.6 before autoclaving. All media for maintenance and experiments were autoclaved at 121 °C and 15 psi (205 kPa) for 25 min. All plant growth regulators were added to the culture media before autoclaving. Culture media were poured into baby food jars (25 mL per jar), and six shoot explants were transferred onto the culture medium in each jar. The experiment was conducted with four replications (jars) per treatment combination. Shoot explants were maintained for 8 weeks with one subculture after 4 weeks. At each subculture, a few millimeters were trimmed from the base of each stem to remove old and dead cells, after which they were placed in fresh medium.

### 5.5. Data Collection

Following a two-month growth period, stem explants were harvested, and data collected. Growth parameters evaluated were the number of axillary shoots formed, the length of the longest shoot on each explant, and shoot dry weight (biomass) for each individual explant. To determine shoot biomass, samples were dried at 70 °C for 72 h and then weighed.

### 5.6. Statistical Analysis

Analysis was completed by using a Response Surface regression (Proc RSREG: 26) with responses as a function of linear, quadratic, and two-way crossproducts of the factors N, Ca, Zeatin, and P. Due to limited response across its concentration range, K entered the model only as a linear covariate. All factor levels were standardized as (Value—mean)/(½xrange) prior to estimation. Standardization, referred to as “coding” in RSM, helped alleviate differences due to changes in component units and magnitude. Separate response surface models were estimated for each response (dependent variables including number of axillary shoots, shoot length, and dry weight). Standard residual diagnostics were used on each model to assess potential outlying values and assure adequate model fit. Following model estimation and assessment, the factor values corresponding to the surface optima (in most cases maximum response) were computed as necessary to give the highest response value and optimum shoot growth.

## 6. Conclusions

This study determined the optimum concentration of selected minerals (N, Ca, and P) and Zea in the culture medium used for axillary shoot production of little-leaf mockorange. Based on response surfaces created by RSM quadratic regression models, the optimum concentrations of Zea, N, Ca, and P were 1.1 µM, 34 to 39 mM, 1.875 mM, and 0.625 mM, respectively. Considering these concentrations and comparing them with standard MS medium, little-leaf mockorange should grow well on ½ strength MS medium supplemented with 1.1 µM Zea for *in vitro* propagation. Using ½ MS strength MS as basal salts will save time and money, as compared to the preparation of a custom nutrient mix.

## Figures and Tables

**Table 1:** Optimum concentrations of Zea, N, Ca, or P resulting in the optimal axillary shoot number, length, and dry weight of little-leaf mockorange shoots produced in tissue culture as determined by RSM models. The concentrations of N, Ca, and P in ½ strength MS medium are shown for comparison.

	Zea (µM)	N (mM)	Ca (mM)	P (mM)
Axillary shoot number	1~1.1	≥37.5	1.75~1.875	0.56~0.625
Shoot length	1.1	32.5	1.75~1.875	0.625
Dry weight	1.1	30~32.5	1.75	0.625
½ MS concentrations	-	30	1.5	0.625

**Table 2:** The treatment design created by SAS software to complete RSM for optimizing *in vitro* growth of little-leaf mockorange shoots. The tested variables included zeatin, N, Ca, P, and K supplied in the culture medium in various concentrations.

Table	Zeatin (µM)	Nitrogen (mM)	Calcium (mM)	Phosphorus (mM)	Potassium (mM)
1	0.82	22.5	1.5	0.625	10
2	0.82	37.5	1.5	0.625	10
3	1.37	22.5	1.5	0.625	10
4	1.37	37.5	1.5	0.625	10
5	1.095	30	1.12	0.31	10
6	1.095	30	1.12	0.937	10
7	1.095	30	1.875	0.31	10
8	1.095	30	1.875	0.937	10
9	1.095	22.5	1.5	0.625	5
10	1.095	22.5	1.5	0.625	15
11	1.095	37.5	1.5	0.625	5
12	1.095	37.5	1.5	0.625	15
13	0.82	30	1.12	0.625	10
14	0.82	30	1.875	0.625	10
15	1.37	30	1.12	0.625	10
16	1.37	30	1.875	0.625	10
17	1.095	30	1.5	0.31	5
18	1.095	30	1.5	0.31	15
19	1.095	30	1.5	0.937	5
20	1.095	30	1.5	0.937	15
21	0.82	30	1.5	0.31	10
22	0.82	30	1.5	0.937	10
23	1.37	30	1.5	0.31	10
24	1.37	30	1.5	0.937	10
25	1.095	22.5	1.12	0.625	10
26	1.095	22.5	1.875	0.625	10
27	1.095	37.5	1.12	0.625	10
28	1.095	37.5	1.875	0.625	10
29	1.095	30	1.12	0.625	5
30	1.095	30	1.12	0.625	15
31	1.095	30	1.875	0.625	5
32	1.095	30	1.875	0.625	15
33	0.82	30	1.5	0.625	5
34	0.82	30	1.5	0.625	15
35	1.37	30	1.5	0.625	5
36	1.37	30	1.5	0.625	15
37	1.095	22.5	1.5	0.31	10
38	1.095	22.5	1.5	0.937	10
39	1.095	37.5	1.5	0.31	10
40	1.095	37.5	1.5	0.937	10
41	1.095	30	1.5	0.625	10
42	1.095	30	1.5	0.625	10
43	1.095	30	1.5	0.625	10
44	1.095	30	1.5	0.625	10
45	1.095	30	1.5	0.625	10
46	1.095	30	1.5	0.625	10

**Table 3:** The high, low, and average concentrations of selected minerals N, Ca, P, and K as well as zeatin, used in different treatment combinations of culture media for *in vitro* propagation of little-leaf mockorange shoots.

Components	Concentrations		
	Low	Medium	High
Zea (µM)	0.82	1.095	1.37
N (mM)	22.5	30	37.5
Ca (mM)	1.125	1.5	1.875
P (mM)	0.31	0.625	0.937
K (mM)	5	10	15

## Data Availability

The raw data supporting the conclusions of this article will be made available by the authors on request.

## Supplementary Materials

The following supporting information can be downloaded at: https://www.mdpi.com/article/10.3390/plants13233446/s1, Figure S1: Explant growth response to various ½ MS (Murashige and Skoog) media formulations. Each jar illustrates a unique treatment condition, with adjustments in nutrient concentrations and/or plant growth regulators. The observed variations in explant growth, vigor, and morphology demonstrate the influence of specific ½ MS media compositions on plant development and health; Table S1: Summary of treatments and their nutrient or components compositions used in ½ strength MS (Murashige and Skoog) medium formulations for different experiments. The table displays the concentrations of macronutrients, micronutrients, organics, vitamins, and PGR (zeatin: cytokinin) along with specific pH values for each treatment; Table S2: Response surface model ANOVA for shoot number; Table S3: Response surface model ANOVA for shoot length; Table S4: Response surface model ANOVA for dry weight; Table S5: Response surface model parameter estimates for shoot number; Table S6: Response surface model parameter estimates for shoot length; Table S7: Response surface model parameter estimates for dry weight.
