# Supplementary material for: Optimization of Selected Minerals and a Cytokinin for In Vitro Propagation of Little-Leaf Mockorange (Philadelphus microphyllus A. Gray) Using Response Surface Methodology (RSM)
Source: Plants (Basel). 2024 Dec 9;13(23):3446. doi: 10.3390/plants13233446 (PMC11644219; doi:10.3390/plants13233446)
Supplement: Supplementary file 1 [file plants-13-03446-s001.zip › plants-3284499-supplementary.pdf]

Table S1. Summary of treatments and their nutrient or components compositions used in ½ strength MS (Murashige and Skoog) medium formulations for different experiments. The table displays the concentrations of macronutrients, micronutrients, organics, vitamins, and PGR (zeatin: cytokinin) along with specific pH values for each treatment.

Treatments (1/2 MS)	1	2	3	4	5	6	7	8	9	10	11
**Macronutrients**	**100 ml**	**100 ml**	**100 ml**	**100 ml**	**100 ml**	**100 ml**	**100 ml**	**100 ml**	**100 ml**	**100 ml**	**100 ml**
NH_4NO_3 (ml)	6.16	8.24	8.24	8.24	8.24	8.24	8.24	8.24	6.16	6.16	6.16
KNO_3 (ml)	7.07	9.5	9.5	4.74	9.5	9.5	9.5	9.5	7.07	7.07	4.4
$CaCl_2$ (ml)	1.66	1.24	1.66	1.66	1.66	1.66	2.48	1.66	1.24	1.66	1.66
KH_2PO_4 (ml)	0.85	0.85	0.424	0.424	0.85	0.85	0.85	0.85	0.85	0.424	0.85
$MgSO_4$ (mg)	9.035	9.035	9.035	9.035	9.035	9.035	9.035	9.035	9.035	9.035	9.035
$NaNO_3$ (ml)	-	-	-	4	-	-	-	6.37	-	-	2.3
K_2SO_4 (ml)	2.03	-	0.27	-	4.35	-	-	-	2.03	2.13	-
NaCl (ml)	-	0.044	-	-	-	-	-	-	-	-	-
NaH_2PO_4 (ml)	-	-	-	4.3	-	4.3	-	-	-	-	-
Total Macro n (ml)	15.7441	19.8341	19.8281	15.06675	20.2541	20.2541	21.0741	20.2541	15.3241	15.3181	13.0741
Total Stock (ml)	17.77	19.878	20.0981	23.366	24.6	24.55	21.074	26.62	17.35	17.4481	15.37
Added stock to 100 ml	82.23	80.122	79.9	76.634	75.3959	75.45	78.926	73.3759	82.6459	82.55	84.6259
**Micronutrients**											
$FeSO_4.7H_2O$ (mg)	1.4	1.4	1.4	1.4	1.4	1.4	1.4	1.4	1.4	1.4	1.4
$Na_2EDTA.2H_2O$ (mg)	1.86	1.86	1.86	1.86	1.86	1.86	1.86	1.86	1.86	1.86	1.86
$MnSO_4.H_2O$ (mg)	0.85	0.85	0.85	0.85	0.85	0.85	0.85	0.85	0.85	0.85	0.85
$ZnSO_4.7H_2O$ (mg)	0.43	0.43	0.43	0.43	0.43	0.43	0.43	0.43	0.43	0.43	0.43
H_3BO_3 (mg)	0.31	0.31	0.31	0.31	0.31	0.31	0.31	0.31	0.31	0.31	0.31
KI (ml)	0.0041	0.0041	0.0041	0.00275	0.0041	0.0041	0.0041	0.0041	0.0041	0.0041	0.0041
$Na_2MoO_4.2H_2O$ (mg)	0.012	0.012	0.012	0.012	0.012	0.012	0.012	0.012	0.012	0.012	0.012
$CuSO_4.5H_2O$ (mg)	0.0012	0.0012	0.0012	0.0012	0.0012	0.0012	0.0012	0.0012	0.0012	0.0012	0.0012
$CoCl.6H_2O$ (mg)	0.0012	0.0012	0.0012	0.0012	0.0012	0.0012	0.0012	0.0012	0.0012	0.0012	0.0012
**PGR (µM)**	0.82	0.82	0.82	0.82	0.82	0.82	0.82	0.82	1.095	1.095	1.095
Zea (µl)	18.2	18.2	18.2	18.2	18.2	18.2	18.2	18.2	24.31	24.31	24.31
**Organics & Vitamins**											
Stock G (ml)	0.5	0.5	0.5	0.5	0.5	0.5	0.5	0.5	0.5	0.5	0.5
Stock H (ml)	0.5	0.5	0.5	0.5	0.5	0.5	0.5	0.5	0.5	0.5	0.5
Sugar (g)	3	3	3	3	3	3	3	3	3	3	3
pH	5.6	5.6	5.6	5.6	5.6	5.6	5.6	5.6	5.6	5.6	5.6

Treatments	12	13	14	15	16	17	18	19	20	21	22	23
Macronutrients	100 ml	100 ml	100 ml	100 ml	100 ml	100 ml	100 ml	100 ml	100 ml	100 ml	100 ml	100 ml
NH_4NO_3 (ml)	6.16	6.16	6.16	8.24	8.24	8.24	8.24	8.24	8.24	8.24	8.24	8.24
KNO_3 (ml)	7.07	7.07	7.07	9.5	4.74	9.5	9.5	4.74	9.5	9.5	9.5	9.5
$CaCl_2$ (ml)	1.66	1.66	2.07	1.24	1.24	1.24	1.24	1.66	1.66	1.66	1.66	1.66
KH_2PO_4 (ml)	0.85	1.27	0.85	0.424	0.424	0.85	0.85	0.424	0.424	0.85	0.85	0.85
$MgSO_4$ (mg)	9.035	9.035	9.035	9.035	9.035	9.035	9.035	9.035	9.035	9.035	9.035	9.035
$NaNO_3$ (ml)	-	-	-	-	4	-	-	4	-	-	-	-
K_2SO_4 (ml)	6.36	1.74	2.03	0.27	-	4.35	-	-	4.61	-	-	-
NaCl (ml)	-	-	-	-	-	-	-	-	-	-	-	-
NaH_2PO_4 (ml)	-	-	-	-	4.3	-	4.3	-	-	-	-	-
Total Macro (m	15.7441	16.1641	16.1541	19.4081	14.64675	19.8341	19.8341	15.0681	19.8281	20.2541	20.2541	20.2541
Total Stock (ml)	22.1	17.9	18.18	19.67	22.946	24.18	24.13	19.068	24.4381	20.2541	20.2541	20.2541
stock to 100 ml	77.9	82.1	81.82	80.32	77.05	75.81	75.87	80.93	75.5619	79.7459	79.7459	79.7459
Micronutrients												
$FeSO_4.7H_2O$ (m	1.4	1.4	1.4	1.4	1.4	1.4	1.4	1.4	1.4	1.4	1.4	1.4
$Na_2EDTA.2H_2O$	1.86	1.86	1.86	1.86	1.86	1.86	1.86	1.86	1.86	1.86	1.86	1.86
$MnSO_4.H_2O$ (m	0.85	0.85	0.85	0.85	0.85	0.85	0.85	0.85	0.85	0.85	0.85	0.85
$ZnSO_4.7H_2O$ (m	0.43	0.43	0.43	0.43	0.43	0.43	0.43	0.43	0.43	0.43	0.43	0.43
H_3BO_3 (mg)	0.31	0.31	0.31	0.31	0.31	0.31	0.31	0.31	0.31	0.31	0.31	0.31
KI (ml)	0.0041	0.0041	0.0041	0.0041	0.00275	0.0041	0.0041	0.0041	0.0041	0.0041	0.0041	0.0041
$Na_2MoO_4.2H_2O$	0.012	0.012	0.012	0.012	0.012	0.012	0.012	0.012	0.012	0.012	0.012	0.012
$CuSO_4.5H_2O$ (m	0.0012	0.0012	0.0012	0.0012	0.0012	0.0012	0.0012	0.0012	0.0012	0.0012	0.0012	0.0012
$CoCl.6H_2O$ (mg)	0.0012	0.0012	0.0012	0.0012	0.0012	0.0012	0.0012	0.0012	0.0012	0.0012	0.0012	0.0012
PGR (μM)	1.095	1.095	1.095	1.095	1.095	1.095	1.095	1.095	1.095	1.095	1.095	1.095
Zea (μl)	24.31	24.31	24.31	24.31	24.31	24.31	24.31	24.31	24.31	24.31	24.31	24.31
Orgs & Vits												
Stock G (ml)	0.5	0.5	0.5	0.5	0.5	0.5	0.5	0.5	0.5	0.5	0.5	0.5
Stock H (ml)	0.5	0.5	0.5	0.5	0.5	0.5	0.5	0.5	0.5	0.5	0.5	0.5
Sugar (g)	3	3	3	3	3	3	3	3	3	3	3	3
pH	5.6	5.6	5.6	5.6	5.6	5.6	5.6	5.6	5.6	5.6	5.6	5.6

Treatments	24	25	26	27	28	29	30	31	32	33	34
Macronutrients	100 ml	100 ml	100 ml	100 ml	100 ml	100 ml	100 ml	100 ml	100 ml	100 ml	100 ml
NH_4NO_3 (ml)	8.24	8.24	8.24	8.24	8.24	8.24	8.24	8.24	8.24	8.24	10.16
KNO_3 (ml)	9.5	9.5	9.5	4.4	9.5	9.5	4.4	9.5	9.5	9.5	9.5
$CaCl_2$ (ml)	1.66	1.66	1.66	1.66	1.66	2.48	2.48	2.48	2.48	2.48	1.66
KH_2PO_4 (ml)	0.85	0.85	0.85	0.85	1.27	0.424	0.85	0.85	0.85	0.85	0.424
$MgSO_4$ (mg)	9.035	9.035	9.035	9.035	9.035	9.035	9.035	9.035	9.035	9.035	9.035
$NaNO_3$ (ml)	-	-	-	4.21	-	-	4.21	-	-	2.25	2.25
K_2SO_4 (ml)	-	-	-	-	4.04	0.021	-	4.35	-	-	0.021
NaCl (ml)	-	-	-	-	-	-	-	-	-	-	-
NaH_2PO_4 (ml)	-	-	-	4.3	-	-	-	-	4.3	-	-
Total Macro (ml)	20.2541	20.2541	20.2541	15.1541	20.6741	20.6481	15.9741	21.0741	21.0741	21.0741	21.7481
Total Stock (ml)	20.2541	20.2541	20.2541	23.66	24.71	20.6691	20.18	25.42	25.37	23.32	24.0191
stock to 100 ml	79.7459	79.7459	79.7459	76.34	75.29	79.33	79.82	74.576	74.6259	76.6759	75.9809
Micronutrients											
$FeSO_4.7H_2O$ (mg)	1.4	1.4	1.4	1.4	1.4	1.4	1.4	1.4	1.4	1.4	1.4
$Na_2EDTA.2H_2O$ (mg)	1.86	1.86	1.86	1.86	1.86	1.86	1.86	1.86	1.86	1.86	1.86
$MnSO_4.H_2O$ (mg)	0.85	0.85	0.85	0.85	0.85	0.85	0.85	0.85	0.85	0.85	0.85
$ZnSO_4.7H_2O$ (mg)	0.43	0.43	0.43	0.43	0.43	0.43	0.43	0.43	0.43	0.43	0.43
H_3BO_3 (mg)	0.31	0.31	0.31	0.31	0.31	0.31	0.31	0.31	0.31	0.31	0.31
KI (ml)	0.0041	0.0041	0.0041	0.0041	0.0041	0.0041	0.0041	0.0041	0.0041	0.0041	0.0041
$Na_2MoO_4.2H_2O$ (mg)	0.012	0.012	0.012	0.012	0.012	0.012	0.012	0.012	0.012	0.012	0.012
$CuSO_4.5H_2O$ (mg)	0.0012	0.0012	0.0012	0.0012	0.0012	0.0012	0.0012	0.0012	0.0012	0.0012	0.0012
$CoCl.6H_2O$ (mg)	0.0012	0.0012	0.0012	0.0012	0.0012	0.0012	0.0012	0.0012	0.0012	0.0012	0.0012
PGR (µM)	1.095	1.095	1.095	1.095	1.095	1.095	1.095	1.095	1.095	1.095	1.095
Zea (µl)	24.31	24.31	24.31	24.31	24.31	24.31	24.31	24.31	24.31	24.31	24.31
Orgs & Vits											
Stock G (ml)	0.5	0.5	0.5	0.5	0.5	0.5	0.5	0.5	0.5	0.5	0.5
Stock H (ml)	0.5	0.5	0.5	0.5	0.5	0.5	0.5	0.5	0.5	0.5	0.5
Sugar (g)	3	3	3	3	3	3	3	3	3	3	3
pH	5.6	5.6	5.6	5.6	5.6	5.6	5.6	5.6	5.6	5.6	5.6

Treatments	35	36	37	38	39	40	41	42	43	44	45	46
Macronutrients	100 ml	100 ml	100 ml	100 ml	100 ml	100 ml	100 ml	100 ml	100 ml	100 ml	100 ml	100 ml
NH_4NO_3 (ml)	11.6	10.16	10.16	10.16	6.16	8.24	8.24	8.24	8.24	8.24	8.24	10.16
KNO_3 (ml)	4.4	12.12	9.5	9.5	7.07	9.5	9.5	4.4	9.5	9.5	9.5	9.5
$CaCl_2$ (ml)	1.66	1.66	1.66	2.48	1.66	1.24	1.66	1.66	1.66	1.66	2.48	1.66
KH_2PO_4 (ml)	0.85	0.85	0.85	0.85	0.85	0.85	0.424	0.85	0.85	0.85	0.85	0.85
$MgSO_4$ (mg)	9.035	9.035	9.035	9.035	9.035	9.035	9.035	9.03	9.035	9.035	9.035	9.035
$NaNO_3$ (ml)	1.37	-	2.25	2.25	-	-	-	3.7	-	-	-	2.25
K_2SO_4 (ml)	-	2.03	-	-	2.03	-	0.27	-	4.35	-	-	-
NaCl (ml)	-	-	-	-	-	-	-		-	-	-	-
NaH_2PO_4 (ml)	-	-	4.3	-	-	-	-	-	-	4.3	-	-
Total Macro (ml)	18.5141	24.7941	22.1741	22.9941	15.7441	19.8341	19.8281	15.1541	20.2541	20.2541	21.0741	22.1741
Total Stock (ml)	19.88	26.82	28.72	25.24	17.77	19.8341	19.55	18.85	24.6	24.5541	21.0741	24.42
stock to 100 ml	80.1159	73.1759	71.2759	74.76	82.2259	80.1659	80.449	81.1459	75.3959	75.4459	78.9259	75.5759
Micronutrients												
$FeSO_4.7H_2O$ (mg)	1.4	1.4	1.4	1.4	1.4	1.4	1.4	1.4	1.4	1.4	1.4	1.4
$Na_2EDTA.2H_2O$ (mg	1.86	1.86	1.86	1.86	1.86	1.86	1.86	1.86	1.86	1.86	1.86	1.86
$MnSO_4.H_2O$ (mg)	0.85	0.85	0.85	0.85	0.85	0.85	0.85	0.85	0.85	0.85	0.85	0.85
$ZnSO_4.7H_2O$ (mg)	0.43	0.43	0.43	0.43	0.43	0.43	0.43	0.43	0.43	0.43	0.43	0.43
H_3BO_3 (mg)	0.31	0.31	0.31	0.31	0.31	0.31	0.31	0.31	0.31	0.31	0.31	0.31
KI (ml)	0.0041	0.0041	0.0041	0.0041	0.0041	0.0041	0.0041	0.0041	0.0041	0.0041	0.0041	0.0041
$Na_2MoO_4.2H_2O$ (m	0.012	0.012	0.012	0.012	0.012	0.012	0.012	0.012	0.012	0.012	0.012	0.012
$CuSO_4.5H_2O$ (mg)	0.0012	0.0012	0.0012	0.0012	0.0012	0.0012	0.0012	0.0012	0.0012	0.0012	0.0012	0.0012
$CoCl.6H_2O$ (mg)	0.0012	0.0012	0.0012	0.0012	0.0012	0.0012	0.0012	0.0012	0.0012	0.0012	0.0012	0.0012
PGR (µM)	1.095	1.095	1.095	1.095	1.37	1.37	1.37	1.37	1.37	1.37	1.37	1.37
Zea (µl)	24.31	24.31	24.31	24.31	30.419	30.419	30.419	30.419	30.419	30.419	30.419	30.419
Orgs & Vits												
Stock G (ml)	0.5	0.5	0.5	0.5	0.5	0.5	0.5	0.5	0.5	0.5	0.5	0.5
Stock H (ml)	0.5	0.5	0.5	0.5	0.5	0.5	0.5	0.5	0.5	0.5	0.5	0.5
Sugar (g)	3	3	3	3	3	3	3	3	3	3	3	3
pH	5.6	5.6	5.6	5.6	5.6	5.6	5.6	5.6	5.6	5.6	5.6	5.6

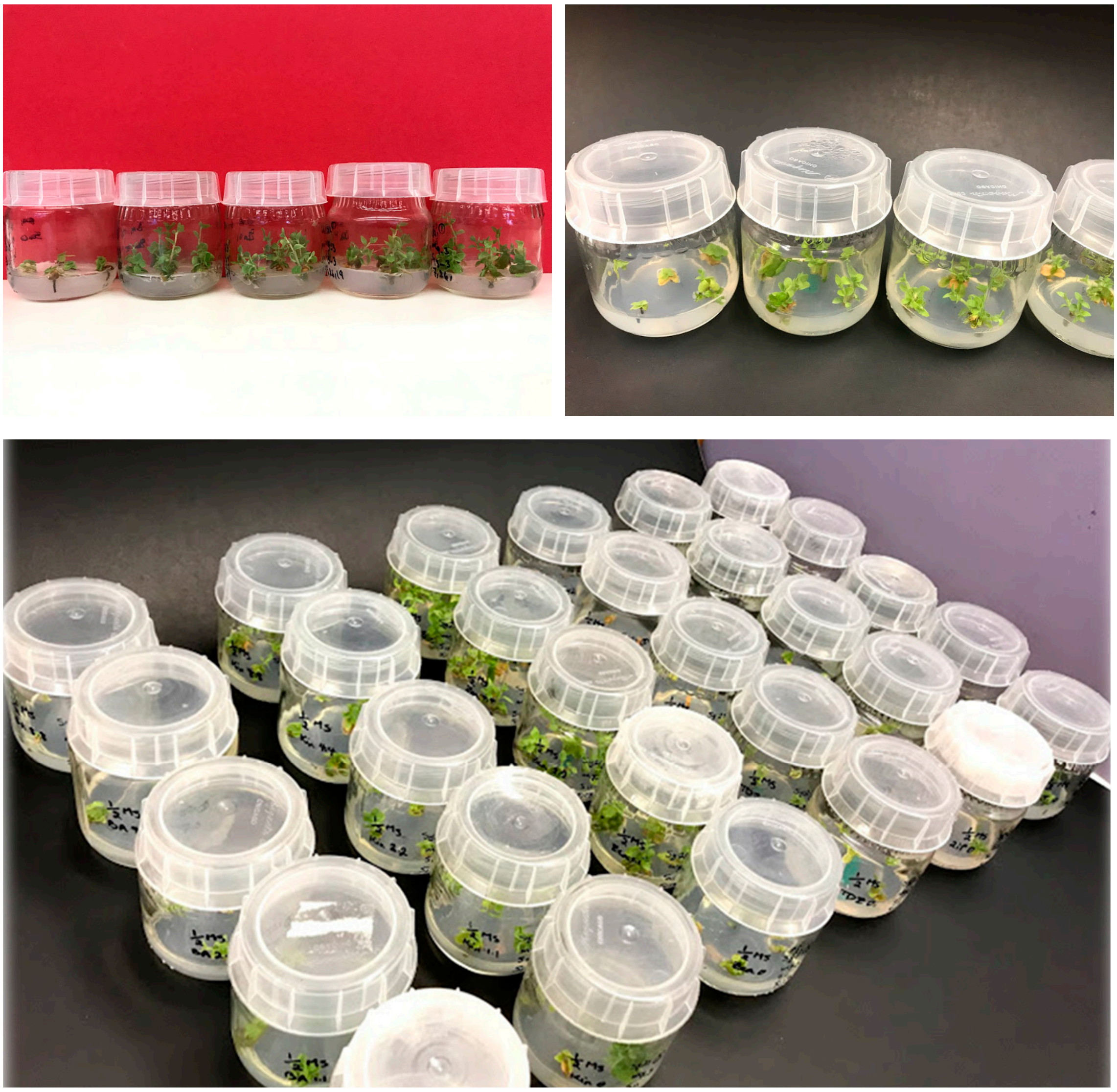

Figure S1. Explant growth response to various ½ MS (Murashige and Skoog) media formulations. Each jar illustrates a unique treatment condition, with adjustments in nutrient concentrations and/or plant growth regulators. The observed variations in explant growth, vigor, and morphology demonstrate the influence of specific ½ MS media compositions on plant development and health.

Table S2. Response surface model ANOVA for shoot number.

Regression	DF	Type I Sum of Squares	R-Square	F Value	Pr > F
**Covariates**	1	0.009946	0.0003	0.05	0.8216
**Linear**	4	2.778791	0.0703	3.56	0.0081
**Quadratic**	4	4.083033	0.1033	5.23	0.0005
**Crossproduct**	6	0.282372	0.0071	0.24	0.9622
**Total Model**	15	7.154142	0.1810	2.45	0.0030

Table S3. Response surface model ANOVA for shoot length.

Regression	DF	Type I Sum of Squares	R-Square	F Value	Pr > F
**Covariates**	1	0.527309	0.0121	2.47	0.1180
**Linear**	4	1.104518	0.0253	1.29	0.2748
**Quadratic**	4	5.651633	0.1295	6.62	<.0001
**Crossproduct**	6	0.918759	0.0210	0.72	0.6363
**Total Model**	15	8.202219	0.1879	2.56	0.0018

Table S4. Response surface model ANOVA for dry weight.

Regression	DF	Type I Sum of Squares	R-Square	F Value	Pr > F
**Covariates**	1	0.004459	0.0045	0.91	0.3412
**Linear**	4	0.019390	0.0196	0.99	0.4143
**Quadratic**	4	0.119422	0.1209	6.10	0.0001
**Crossproduct**	6	0.022185	0.0225	0.76	0.6059
**Total Model**	15	0.165457	0.1675	2.25	0.0065

Table S5. Response surface model parameter estimates for shoot number.

Parameter	DF	Estimate	Standard Error	t Value	Pr > |t|	Parameter Estimate from Coded Data
**Intercept**	1	-6.023576	4.222478	-1.43	0.1556	1.875820
**N**	1	0.045609	0.058788	0.78	0.4390	0.126114
**Ca**	1	0.726967	1.174560	0.62	0.5368	0.102425
**Zeatin**	1	6.564790	3.196774	2.05	0.0416	-0.105132
**P**	1	2.682570	1.309471	2.05	0.0421	-0.077912
**N*N**	1	-0.000241	0.000323	-0.74	0.4574	-0.054133
**Ca*N**	1	-0.001129	0.010157	-0.11	0.9116	-0.012706
**Ca*Ca**	1	-0.052491	0.129201	-0.41	0.6851	-0.029526
**Zeatin*N**	1	0.001383	0.026763	0.05	0.9589	0.005703
**Zeatin*Ca**	1	0.103206	0.535266	0.19	0.8473	0.021286
**Zeatin*Zeatin**	1	-3.303946	0.955337	-3.46	0.0007	-0.249861
**P*N**	1	-0.005165	0.011776	-0.44	0.6615	-0.048420
**P*Ca**	1	-0.256558	0.235517	-1.09	0.2776	-0.120262
**P*Zeatin**	1	-0.083215	0.642319	-0.13	0.8971	-0.014303
**P*P**	1	-0.654618	0.184953	-3.54	0.0005	-0.255710
**K**	1	-0.001247	0.005520	-0.23	0.8216	-0.001247

Table S6. Response surface model parameter estimates for shoot length.

Parameter	DF	Estimate	Standard Error	t Value	Pr > |t|	Parameter Estimate from Coded Data
**Intercept**	1	-9.885361	4.418397	-2.24	0.0266	1.379966
**N**	1	0.067438	0.061515	1.10	0.2745	0.089374
**Ca**	1	1.295560	1.229059	1.05	0.2934	0.053045
**Zeatin**	1	8.411983	3.345101	2.51	0.0129	-0.077002
**P**	1	4.064347	1.370229	2.97	0.0035	0.021529
**N*N**	1	-0.000646	0.000338	-1.91	0.0577	-0.145317
**Ca*N**	1	0.010295	0.010629	0.97	0.3342	0.115816
**Ca*Ca**	1	-0.196383	0.135196	-1.45	0.1482	-0.110466
**Zeatin*N**	1	0.001375	0.028005	0.05	0.9609	0.005673
**Zeatin*Ca**	1	-0.200447	0.560102	-0.36	0.7209	-0.041342
**Zeatin*Zeatin**	1	-3.676595	0.999664	-3.68	0.0003	-0.278043
**P*N**	1	-0.013094	0.012322	-1.06	0.2895	-0.122756
**P*Ca**	1	-0.355782	0.246445	-1.44	0.1507	-0.166773
**P*Zeatin**	1	-0.097140	0.672122	-0.14	0.8853	-0.016696
**P*P**	1	-0.828218	0.193535	-4.28	<.0001	-0.323523
**K**	1	-0.009077	0.005776	-1.57	0.1180	-0.009077

Table S7. Response surface model parameter estimates for dry weight.

Parameter	DF	Estimate	Standard Error	t Value	Pr > |t|	Parameter Estimate from Coded Data
**Intercept**	1	-1.622966	0.665601	-2.44	0.0158	0.213012
**N**	1	0.015592	0.009274	1.68	0.0946	0.012367
**Ca**	1	0.178355	0.185473	0.96	0.3376	0.008744
**Zeatin**	1	1.374083	0.505432	2.72	0.0072	-0.008533
**P**	1	0.518414	0.207333	2.50	0.0134	-0.000875
**N*N**	1	-0.000116	0.000050631	-2.30	0.0229	-0.026159
**Ca*N**	1	0.000712	0.001555	0.46	0.6477	0.008006
**Ca*Ca**	1	-0.022486	0.020252	-1.11	0.2685	-0.012648
**Zeatin*N**	1	-0.002015	0.004240	-0.48	0.6352	-0.008312
**Zeatin*Ca**	1	0.016636	0.084800	0.20	0.8447	0.003431
**Zeatin*Zeatin**	1	-0.604576	0.150637	-4.01	<.0001	-0.045721
**P*N**	1	-0.000596	0.001866	-0.32	0.7498	-0.005588
**P*Ca**	1	-0.074160	0.037312	-1.99	0.0485	-0.034763
**P*Zeatin**	1	-0.008073	0.101760	-0.08	0.9369	-0.001387
**P*P**	1	-0.101094	0.029163	-3.47	0.0007	-0.039490
**K**	1	-0.000835	0.000875	-0.95	0.3412	-0.000835
